# The Safety of the Patient in Anæsthesia

**Published:** 1909-09

**Authors:** John Freeman

**Affiliations:** Anæsthetist to the Bristol General Hospital and the Orthopaedic Hospital


### THE SAFETY OF THE PATIENT IN ANÆSTHESIA.

BY

JOHN FREEMAN, M.D.Durh., F.R.C.S.Edin.

*Anæsthetist to the Bristol General Hospital and the Orthopaedic Hospital.*

THE remarks recently made by coroners when holding inquests in cases where death had occurred during anæsthesia require the serious attention of the medical profession, and as I have now administered anæsthetics in several thousand cases, my experience with respect to the choice of the anæsthetic may be of interest.

When I commenced giving anæsthetics at the General Hospital in 1891 chloroform was the routine anæsthetic. I was not satisfied, however, with the signs some of the patients showed when under its influence, and I therefore began to substitute ether. In 1894 experience confirmed my opinion. I was asked to give an anæsthetic to a boy aged seven for the operation of circumcision, and I used chloroform. In the middle of the operation a deathly pallor suddenly came over him, his respiration failed, and he was pulseless. For several minutes he was in a desperate condition, but fortunately, after a time of great anxiety, we were able to revive him. This case led me to the conclusion that chloroform was not such a safe anæsthetic for children as was then supposed.

Since that time much evidence of a similar nature has accumulated. Professor Waller has demonstrated how much more poisonous chloroform is than ether on nerve tissue, and many inexplicable deaths have occurred under chloroform in the hands of experienced anæsthetists, the result of which is that the majority of anæsthetists now rely more upon ether than upon chloroform. Dr. Luke,[1] the instructor of anæsthetics at the Edinburgh Royal Infirmary, in his book on anæsthetics gives the relative mortality under various agents. "Ether, 1 in 10,000; ACE and CE (one part chloroform and two parts ether) mixtures, 1 in 7,500; chloroform, at least 1 in 1,000." These are striking figures coming from such an authority.

I am well aware that there are many chloroformists who are so skilful that they do not appear to produce dangerous symptoms with their patients, and consequently look upon chloroform as a safe anæsthetic, but it is often the unexpected which happens. I know an eminent specialist who for several years had chloroform given for his operations. One day he had the misfortune to lose a patient under it, and since then he has used ether.

In midwifery practice the question of the anæsthetic is exceptional. The woman in labour is different in several respects from a woman about to undergo an ordinary operation. It has been found that whiffs of chloroform are practically safe for her, but should the case go on to operative midwifery, those who have had large experience in this kind of work consider that it is safer to change to ether.

The main objection to ether is that it occasionally is followed by bronchial or lung complications. From the fact that it has become the routine anæsthetic in many hospitals, I think the danger cannot have been much in evidence, at any rate from a mortality point of view. An objection, equally great, is now

[1] Luke, *Guide to Anæsthetics.*

made to the use of chloroform in those cases known as delayed chloroform poisoning.

There is no doubt whatever that a patient shows more vitality under ether than under chloroform. In abdominal surgery, some of this vitality—I refer to the increased respiratory movements—occasionally interferes with the manipulations of the operator, and then the more lethal chloroform has to be used to tranquilise the respiration and to relax the muscles.

The public mind has recently been agitated by the condition known as status lymphaticus in connection with anæsthesia. In every case that I have happened to read chloroform was used. It is a question whether ether, with its stimulating effect, would not be a safer anæsthetic to employ with the type of patient in which this condition is found. It is always sad to see a patient, who was in a serious condition before the operation was commenced, die on the table ; but when a healthy person dies from the anæsthetic, administered perhaps for some small operation, such as the extraction of teeth, the result is one of the greatest tragedies a medical man can meet with.

To recently-qualified men who as students were taught how to use ether my advice is that they should make it their routine surgical anæsthetic. To those who are not recently qualified, I should like to suggest, after long experience, that the safety of the patient is greater when the anæsthesia is produced by ACE or CE than when produced by chloroform alone.

